# The association between ballroom dance training and empathic concern: Behavioral and brain evidence

**DOI:** 10.1002/hbm.26042

**Published:** 2022-08-16

**Authors:** Xiao Wu, Xuejing Lu, Huijuan Zhang, Xiao Wang, Yazhuo Kong, Li Hu

**Affiliations:** ^1^ CAS Key Laboratory of Behavioral Science Institute of Psychology Beijing China; ^2^ Department of Psychology University of Chinese Academy of Sciences Beijing China; ^3^ CAS Key Laboratory of Mental Health Institute of Psychology Beijing China; ^4^ School of Art Beijing Sport University Beijing China

**Keywords:** anterior cingulate cortex, ballroom dance, empathic concern, occipital gyrus, resting‐state functional connectivity, trait empathy

## Abstract

Dance is unique in that it is a sport and an art simultaneously. Beyond improving sensorimotor functions, dance training could benefit high‐level emotional and cognitive functions. Duo dances also confer the possibility for dancers to develop the abilities to recognize, understand, and share the thoughts and feelings of their dance partners during the long‐term dance training. To test this possibility, we collected high‐resolution structural and resting‐state functional magnetic resonance imaging (MRI) data from 43 expert‐level ballroom dancers (a model of long‐term exposure to duo dance training) and 40 age‐matched and sex‐matched nondancers, and measured their empathic ability using a self‐report trait empathy scale. We found that ballroom dancers showed higher scores of empathic concern (EC) than controls. The EC scores were positively correlated with years with dance partners but negatively correlated with the number of dance partners for ballroom dancers. These behavioral results were supported by the structural and functional MRI data. Structurally, we observed that the gray matter volumes in the subgenual anterior cingulate cortex (ACC) and EC scores were positively correlated. Functionally, the connectivity between ACC and occipital gyrus was positively correlated with both EC scores and years with dance partners. In addition, the relationship between years with dance partners and EC scores was indirect‐only mediated by the ACC‐occipital gyrus functional connectivity. Therefore, our findings provided solid evidence for the close link between long‐term ballroom dance training and empathy, which deepens our understanding of the neural mechanisms underlying this phenomenon.

## Graphical abstract

This study provided solid behavioral and neural evidence showing that long‐term ballroom dance training with relatively fixed dance partners is associated with one's empathic concern (EC). Theoretically, this study deepens our understanding of the neural mechanisms underlying the link between ballroom dance training and EC, and highlights the crucial role of resting‐state functional connectivity between the anterior cingulate cortex and occipital gyrus in mediating the relationship between dance training and EC. Practically, this study shed new insight into the development of duo dance‐based programs to improve empathic ability, thus helping people with impaired empathy, such as individuals with schizophrenia or autism spectrum disorder.

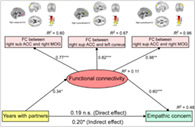

## 1 INTRODUCTION

Dance exists in every culture as a special form of sport and art. Millions of people dance either recreatively or professionally. A range of sensorimotor skills are well‐developed through intensive dancing practice, involving complex sensorimotor abilities (Thullier & Moufti, 2004) and couplings between perception and action (Blasing et al., 2012). When compared with other forms of physical activities, dance training is not only effective in improving sensorimotor functions (Lu et al., 2018) but also benefits high‐level emotional and cognitive functions (Rehfeld et al., 2018). Specifically, some dances have the advantage of combining many diverse features, including physical activity, emotional communication, and social interaction, compared with repetitive physical activities, such as exercising, walking, or playing an instrument (Kattenstroth et al., 2010). Moreover, dances, especially those involving two or more dancers, also confer the possibility for dancers to develop the abilities to recognize, understand, and share their dance partners' thoughts and feelings, that is, empathy.

By definition, empathy is the capacity to understand or feel what another person is experiencing within their frame of reference (Ren et al., 2020; Zaki & Ochsner, 2012). Therefore, when witnessing the action, sensation, and emotions of others, brain regions that involve in one's own action, sensation, and emotion are consistently activated, including the anterior insula, prefrontal cortex, anterior and middle cingulate cortex, and temporoparietal junction (de Waal & Preston, 2017; Kogler et al., 2020; Lamm et al., 2019). As suggested by the Russian‐doll model, empathy is built upon a perception‐action mechanism, which allows for basic motor mimicry and emotional contagion, and further develops other components of empathy, such as empathic concern (EC) and perspective taking (PT; de Waal & Preston, 2017). The experience of motor mimicry and emotional contagion is highly associated with the mirror neuron system (Iacoboni, 2009), through which induces a similar emotional state in the observer as in the observee. Although empathy emerges and develops early in life, it is flexible and amenable over the lifespan (Decety, 2015), and can be affected by both intraindividual contributors and social factors (McDonald and Messinger, 2011; Wu & Lu, 2021). Since the mirror neurons come from sensorimotor experience, and much of this experience is obtained through interaction with others (Heyes, 2010), it is reasonable to assume physical activities involving imitation and interaction could facilitate the development of empathy.

Indeed, in dances involving the interaction of different dancers (e.g., the duo dance), each dancer is not only a performer but also a spectator observing the action of his/her dance partner. Therefore, such a dance form requires dancers to tightly coordinate with their dance partners in body movements and synchronize with their emotional state. From this perspective, it makes perfect sense to assume that duo dancers exhibit higher empathic responses due to their interaction during the long‐term dance training. Surprisingly, this hypothesis is barely tested, and its possible neural mechanisms are still unclear.

To examine the relationship between dance training and empathic ability and its neural basis, we recruited 43 actively performing, expert‐level ballroom dancers, as a model of long‐term exposure to duo dance training, and 40 age‐matched and sex‐matched nondancers. The difference in empathic ability between dancers and nondancers was quantified using a self‐report measure of the Interpersonal Reactivity Index (IRI; Davis, 1980). Please note that the IRI that measures both affective and cognitive empathy is one of the most popular questionnaires to measure empathy (Ren et al., 2019) and has been well validated in the Chinese population (Zhao et al., 2018). The brain structural and functional data were assessed using high‐resolution T1‐weighted structural and resting‐state functional magnetic resonance imaging (MRI) data, respectively. The relationships among dance training, empathic ability, and brain structure and function were explored using a series of correlation analyses. Based on the correlation results, we built a theoretical modal to quantify their mediation relationship using structural equation modeling. As such, this study would deepen our understanding of the neural mechanisms underlying the association between empathy and ballroom dance training. It should be noted that as a multifaceted construct, empathy varies from person to person (Coll et al., 2017). For instance, neurotic individuals are more prone to experience personal distress (PD; Guilera et al., 2019), while individuals with higher intimate relationship satisfaction are likely to have stronger empathic ability (Wen et al., 2022). Therefore, to rule out the possibility that any group difference in empathy comes from inherent differences between the two groups, such as personality, sociability, and romantic relationship, we also quantified these related variables using questionnaires that tap into these issues.

## 2 MATERIALS AND METHODS

### 2.1 Participants

A total of 83 healthy right‐handed participants from Beijing Sport University were recruited in this study, including 43 professional ballroom dancers and 40 nondancers. To determine the required sample sizes for sufficient power to detect the group difference in empathy, a priori power analysis was conducted using G*power software (version 3.1) with a significant level at .05 by setting statistical power at 0.8 with a medium effect, which yields the minimum sample size of 34 for each group. Two dancers were excluded due to incomplete MRI data collection, yielding a final sample of 81 participants (41 dancers and 40 nondancers, Table 1). The inclusion criteria of dancer group contain: (1) being listed as ballroom dancers by the Chinese Dance Sport Federation; (2) being majored in ballroom dance in college; (3) having ballroom dance experience at least for 5 years; (4) having participated in professional national dance sport competition at least once; (5) practicing more than 10 h/week in the past 3 years. In contrast, the control group had no history of any professional dance training. All included participants had no safety contraindications for MRI, no history of neurological disorders, and did not take any coffee or alcohol within the 24 h before participating in the experiment. This study was approved by the Ethics Committee of the Institute of Psychology, Chinese Academy of Sciences. Written informed consent was obtained from each participant before the experiment, and all participants received monetary compensation after their participation.

**TABLE 1:** Descriptive statistics (*M* ± SD) of demographic information, IRI scores, and dance attributes for dancers and controls

Variables	Dancer group (*n* = 41)	Control group (*n* = 40)	U/t	p value	Cohen's *d*
Sex (male/female)	21/20	20/20	‐	‐	‐
Age (years)	21.78 ± 2.36	20.95 ± 2.14	997.50	.089	‐
Education (years)	15.15 ± 2.03	14.63 ± 1.63	934.00	.271	‐
IRI					
Perspective taking	18.37 ± 4.33	18.58 ± 2.76	−0.26	.797	‐
Empathic concern	20.78 ± 3.71	18.83 ± 3.31	2.50	.014*	0.55
Personal distress	12.88 ± 4.48	13.58 ± 4.19	−0.72	.472	‐
Years of dance	8.93 ± 2.21	‐	‐	‐	‐
Years with dance partners	8.33 ± 1.84	‐	‐	‐	‐
Starting age of dancing	10.61 ± 2.82	‐	‐	‐	‐
Number of dance partners	3.17 ± 1.48	‐	‐	‐	‐

*Notes*: Years of dance refer to the duration of dance training for dancers, including basic training without a fixed dance partner and official training with a fixed dancer partner. Years with dance partners refer to the number of years that the dancer has officially danced with a fixed partner. The number of dance partners refers to the number of fixed partners with which the dancer has danced. *p < .05.

Abbreviations: IRI, Interpersonal Reactivity Index; SD, standard deviation.

### 2.2 Demographic information, trait empathy, and dance attributes

For all participants, basic demographic information, including sex, age, year of education, and daily exercise was collected to ensure that dancers and controls were well‐matched. To evaluate trait empathy, all participants were instructed to complete the Chinese version of IRI before image acquisition. It includes 28 items on a 5‐point Likert Scale (0 = strongly disagree, 4 = strongly agree) and is divided into four subscales: PT, fantasy (FS), EC, and PD. Only PT, EC, and PD were used in this study since FS subscale is limited to fictional stories (Nomura & Akai, 2012). For dancers, we also collected some important dance attributes, including the years of dance (i.e., the duration of dance training, including basic training without a fixed dance partner and official training with a fixed dance partner), years with dance partners (i.e., the number of years that the dancer has officially danced with a fixed dance partner), age started dancing (i.e., the age that the dancer officially started ballroom dance), and number of dance partners (i.e., the number of fixed dance partners that the dancer has danced with).

To rule out the possibility that any group difference in empathy comes from inherent differences between the two groups, we reached all participants on the other day after the original data collection. They were asked to provide information about their romantic relationship at the previous experimental date through an online questionnaire. In addition, participants' personality and sociability were assessed using the Big 5 Inventory‐2 (Zhang et al., 2022) and the Questionnaire of Interpersonal Competence (Wei, 2005). The Big 5 Inventory‐2 consists of 60 items and measures the personality traits from five dimensions: Extraversion, Agreeableness, Conscientiousness, Negative emotionality, and Open‐mindedness. It also includes a “sociability” sub‐dimension in the Extraversion dimension. The Questionnaire of Interpersonal Competence includes 40 items and 5 dimensions: Initiating relationship, Asserting displeasure with others' actions, Self‐disclosure, Managing interpersonal conflicts, and Providing emotional support.

### 2.3 MRI data acquisition

MRI data were collected using a 3.0‐T MRI system (Discovery MR 750; General Electric Healthcare, Milwaukee, WI) at the Magnetic Resonance Imaging Research Center, Institute of Psychology, Chinese Academy of Sciences, China. A standard birdcage with an eight‐channel head coil, along with restraining foam pads and earbuds, was used to minimize head motion and scanner noise. First, high‐resolution T1‐weighted structural images were acquired using a gradient echo (3D SPGR) sequence with the following parameters: repetition time (TR) = 6.7 ms, echo time (TE) = 2.9 ms, flip angle (FA) = 12°, field of view (FOV) = 256 × 256 mm^2^, matrix = 256 × 256, in‐plane resolution = 1 × 1 mm, slice thickness = 1 mm. Second, resting‐state functional images were obtained using echo‐planar imaging sequence with the following parameters: TR = 2000 ms, TE = 30 ms, FA = 90°, FOV = 224 × 224 mm^2^, matrix = 64 × 64, in‐plane resolution = 3 × 3 mm, slice thickness = 3.5 mm, slices = 37, interleaved slice ordering. During the scan, participants were asked to relax and keep their heads still with eyes closed but not fall asleep. Please noted that all participants underwent a functional MRI (fMRI) task after the resting‐state fMRI scan, in which they were passively viewing ballroom dancing movies or natural scenery movies. This task was independent of this study, thus it was not analyzed and reported here.

### 2.4 MRI preprocessing

Structural MRI images were analyzed using Computational Anatomy Toolbox (CAT12; http://dbm.neuro.uni-jena.de/cat12/) in Statistical Parametric Mapping (SPM12; http://www.fil.ion.ucl.ac.uk/spm/software/spm12/) and the results were visualized using xjView toolbox (https://www.alivelearn.net/xjview). The preprocessing steps included denoising, skull stripping, segmentation into gray matter, white matter, and cerebrospinal fluid (CSF), and normalization to Montreal Neurological Institute (MNI) space. During the segmentation, the ICBM space template based on East Asian brains was used in affine regularization, Dartel Registration with default template obtained by standard Dartel registration of 555 IXI subjects between 20 and 80 years was used for spatial registration. The quality of images was assessed with the built‐in image‐quality rating and visually checked. According to a well‐recognized meta‐analysis on empathy network (Fan et al., 2011), a series of empathy‐related regions of interest (ROIs) in the AAL3 atlas (Rolls et al., 2020) were defined in this study. These ROIs included the left and right supplementary motor area, insula, and anterior cingulate cortex (ACC). Based on the AAL3 template, the ACC in each hemisphere was divided into three parts, that is, the subgenual, pregenual, and subgenual ACC. Voxel‐wise estimation of gray matter volume (GMV) in all defined ROIs in the AAL3 atlas (Rolls et al., 2020) was extracted from the preprocessed structural MRI images for each participant for further statistical analyses.

Resting‐state functional images were preprocessed using Data Processing Assistant for resting‐state fMRI (Yan & Zang, 2010) with the standard resting‐state functional connectivity (FC) preprocessing pipeline: removed the first 10 volumes; slice timing with the 37^th^ slice as the reference slice; regressed out nuisance regressors including Friston 24 head motion parameters, white matter signals, and CSF signals; regressed out an overall linear trend; co‐registered T1 images to functional images; segmented T1 images through the Diffeomorphic Anatomical Registration using Exponentiated Lie algebra (DARTEL; Ashburner, 2007) method and chose East Asian template for affine regularization; normalized functional images to the standard MNI space and resampled voxel size of 3 × 3 × 3 mm^3^ by DARTEL method; filtered with a bandpass of 0.01–0.1 Hz; and smoothed with a 4‐mm full width half maximum Gaussian kernel. All participants' maximal head motions were within 2.5 mm translation (in any direction of x, y, or z) and 2.5° rotation throughout scanning. The mean framewise displacements (FD) of all participants were within 0.2 mm. Voxel‐wise FC analysis was conducted to calculate the correlation coefficients between the mean time series within each ROI and other voxels across the whole brain. Finally, the correlation coefficients were transformed to z‐statistic maps using Fisher's *r* to z transform to allow for between‐subject comparisons.

### 2.5 Statistical analyses

#### 2.5.1 Statistical analyses for behavioral data

Before performing statistical analyses, Shapiro–Wilk tests were conducted to evaluate the normality of the data (i.e., demographic information, IRI subscales scores, romantic relationship information, and subscale scores in the Big 5 Inventory‐2 and the Questionnaire of Interpersonal Competence). Results showed that age, year of education, and romantic relationship information were skewed distributed (all p < .05). Nonparametric Mann–Whitney *U* tests were performed to test whether there were differences in these variables between the two groups. Subscale scores of the IRI, Big 5 Inventory‐2, and Questionnaire of Interpersonal Competence were normally distributed (all p > .05). Parametric independent‐samples *t* tests were used to assess group differences in these subscale scores.

Correlation analyses were conducted to examine the relationship between the IRI subscales and dance attributes for all dancers. Since years of dance, years with dance partners, and the number of dance partners were skewed distributed (all p < .05), Spearman rank correlation analyses were conducted with regard to them. In contrast, Pearson correlation analysis was conducted for dancers when the starting age of dancing was involved. To validate the significant correlations, we also performed partial correlation analyses for dancers using age and sex as covariates. Other collected variables, for example, romantic relationship information, were also considered as covariates, whenever necessary.

The above statistical analyses were conducted using SPSS 25.0 (SPSS Inc., Chicago, IL). To account for multiple comparisons in the analyses, a false discovery rate (FDR) procedure (Benjamini & Hochberg, 1995) was adopted to adjust the p values. All tests were two‐tailed, and the level of significance was p = .05.

#### 2.5.2 Statistical analyses for MRI data

For structural MRI data, partial correlation analyses were separately conducted for dancers and controls to examine the relationship between GMV in each ROI and behavioral variables (i.e., IRI subscales and dance attributes), with total intracranial volume (TIV), age, and sex as covariates.

For resting‐state fMRI data, partial correlation analyses were separately conducted for dancers and controls to examine the relationship between the ROI‐based FCs and IRI subscales showing significant group differences with mean FD, age, and sex as covariates. Clusters were considered as significant only if they reached a threshold of voxel‐level p < .001 and cluster‐level family‐wise error‐corrected p < .05. To test whether the significant ROI‐based FCs were also correlated with dance attributes, the corresponding FC values were extracted and correlated with dance attributes using partial correlation analyses, with mean FD, age, and sex as covariates. To validate the significant correlations, other collected variables (e.g., romantic relationship information) were also considered as covariates whenever necessary in the partial correlation analyses. To account for multiple comparisons in the analyses, the FDR procedure was adopted to adjust the p values.

To explore the influencing factors of the relationship between dance attributes and trait empathy, we built a mediation model based on the significant results in the above statistical analyses. The adequacy of the proposed mediation model was tested using structural equation modeling (SEM; Byrne, 2016) based on maximum likelihood estimation. Specifically, years with dance partners were used as the independent variable, EC scores were used as the dependent variable, and resting‐state FCs were used as the mediator variable. The model fit was assessed using the following criteria: the significance of chi‐square (χ^2^) statistic (p value) > .05, the ratio of chi‐square to degrees of freedom (χ^2^/df) < 2 (Kline, 2016), the root mean square error of approximation (RMSEA) ≤ 0.06 (Hu & Bentler, 1999), both the goodness‐of‐fit index (GFI) and the adjusted GFI (AGFI) ≥ 0.90, both the comparative fit index (CFI) and the normed fit index (NFI) ≥ 0.95 (Hooper et al., 2008). Besides, to assess the significance of the indirect and direct effects in the mediation modal, bias‐corrected 95% confidence intervals (CIs) were calculated using the bootstrapping procedure (Preacher & Hayes, 2008). The estimate was considered statistically significant when the 95% CI (based on 2000 bootstrap samples) excluded zero. Standardized estimate (*b*), standard error (SE), 95% CI, and p value were reported for both direct and indirect effects. There are three patterns of mediating effect: (a) a complementary mediation exhibits both significant indirect and direct effects, which point to the same (positive or negative) direction; (b) a competitive mediation exhibits both significant indirect and direct effects, which point to opposite directions; and (c) an indirect‐only mediation exhibits a significant indirect effect but insignificant direct effect (Carrión et al., 2017). These statistical analyses were performed using Amos 24.0 (SPSS Inc., Chicago, IL).

## 3 RESULTS

### 3.1 Behavioral results

**FIGURE 1:**
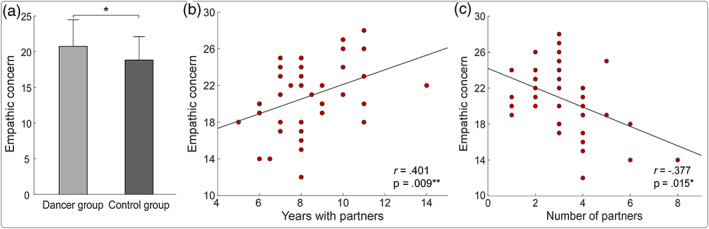
The comparison of EC scores between dancers and controls and correlations between dance attributes and EC scores for dancers. (a) EC score was significantly higher for dancers than controls. (b) Years with dance partners were positively correlated with EC scores. (c) The number of dance partners was negatively correlated with EC scores. EC, empathic concern; *p < .05; **p < .01.

Descriptive statistics of demographic information, IRI scores, and dance attributes expressed as mean ± SD were summarized in Table 1. There were no significant differences in age (21.78 ± 2.36 vs. 20.95 ± 2.14, p = .089) or education level (15.15 ± 2.03 vs. 14.63 ± 1.63, p = .271) between dancers and controls. However, significant difference was observed in EC scores between dancers and controls (20.78 ± 3.71 vs. 18.83 ± 3.31, p = .014; Table 1 and Figure 1). In contrast, there were no significant group differences in PT scores (18.37 ± 4.33 vs. 18.58 ± 2.76, p = .797) or PD scores (12.88 ± 4.48 vs. 13.58 ± 4.19, p = .472). For dancers, years with dance partners were positively correlated with EC scores (*r* = .401, p = .009), and the number of dance partners was negatively correlated with EC scores (*r* = −.377, p = .015; Figure 1). Additionally, the starting age of dancing was negatively correlated with PT scores (*r* = −.336, p = .032). However, this negative correlation did not survive after controlling age and sex (*r* = −.292, p = .071).

Descriptive statistics of daily exercise information and romantic relationship information were summarized in Table S1. Whereas there was no significant difference in daily exercise information (i.e., like sedentary/engage in activities) between dancers and controls (p = .413), significant difference was observed in the current romantic status between dancers and controls (Table S1). More participants in the dancer group than in the control group (26 vs. 13) were in a romantic relationship. Other information about the romantic relationship (i.e., the number of romantic relationships ever had, years of the longest romantic relationship, and the quality of romantic relationships) were significantly larger in the dance group than in the control group (all p < .05; Table S1). For dancers, no significant correlations were observed between EC scores and romantic relationship variables (the number of romantic relationships: *r* = .066, p = .697; years of the longest romantic relationship: *r* = .147, p = .391; quality of romantic relationships: *r* = .128, p = .436). These results suggested that the enhanced EC scores observed in the dancer group could be independent of one's romantic relationship. To directly assess whether the relationship between years with dance partners and EC scores could be influenced by romantic relationships, we also conducted a partial correlation analysis between years with dance partners and EC scores in the dancer group using age, sex, and romantic relationship variables as covariates. The result showed that EC scores were still significantly correlated with years with dance partners (*r* = .419, p = .021).

Descriptive statistics of subscale scores of the Big 5 Inventory‐2 and the Questionnaire of Interpersonal Competence were summarized in Table S2, and no significant group differences for all subscale scores (all p > .05). For dancers, no significant correlations were observed between EC scores and all subscale scores (all p > .05; Table S3). To directly assess whether the relationship between years with dance partners and EC scores could be influenced by personality and sociability factors, we also conducted a partial correlation analysis between years with dance partners and EC scores in the dancer group using age, sex, and subscale scores of the two questionnaires as covariates. The result showed that EC scores were robustly and significantly correlated with years with dance partners (*r* = .454, p = .013).

### 3.2 Correlations between GMV in empathy‐related ROIs and behavioral variables

**FIGURE 2:**
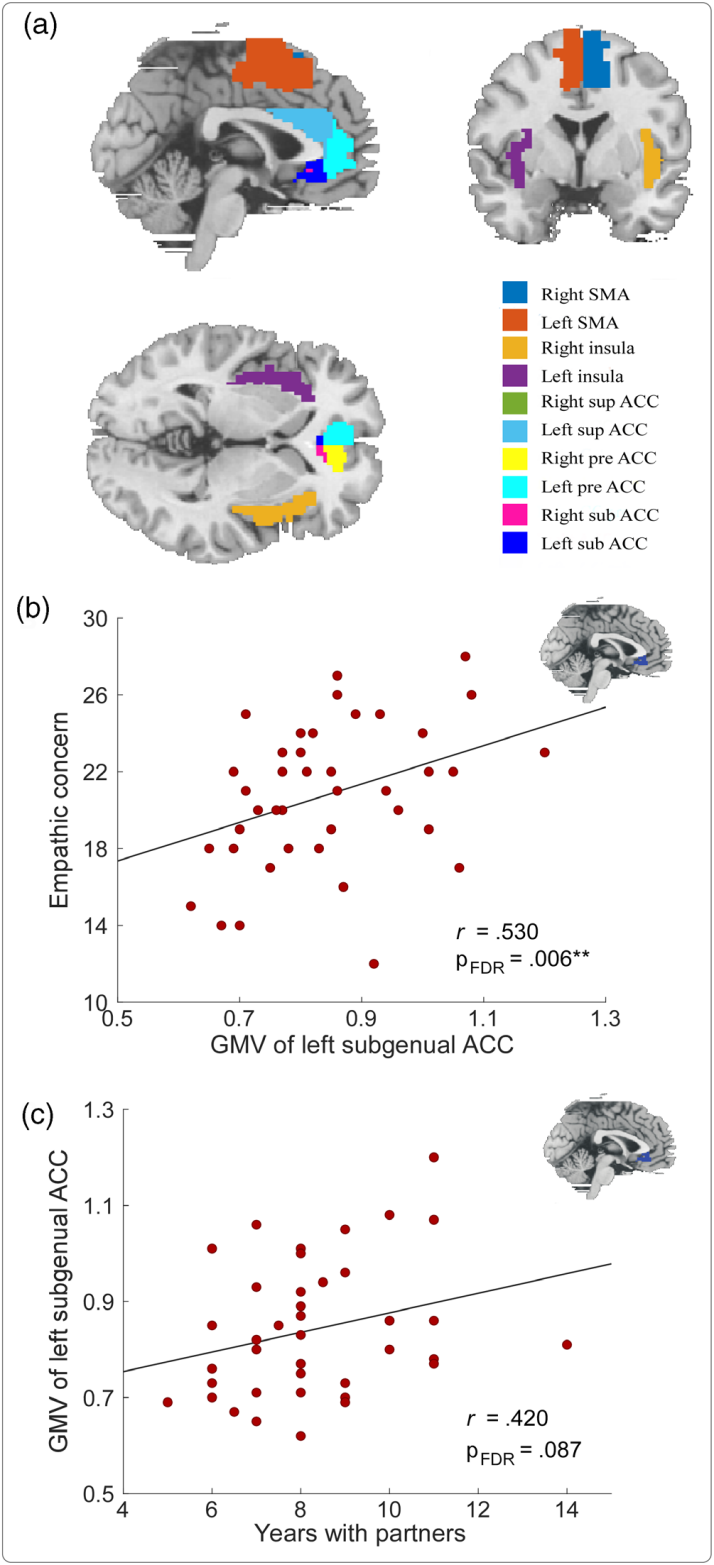
Correlations between GMV of empathy‐related ROIs and years with dance partners or EC scores for dancers. (a) Structural diagram of empathy‐related ROIs. (b) GMV of the left subgenual ACC showed a strong correlation with EC scores. (c) GMV of the left subgenual ACC showed a marginally significant correlation with years with dance partners. TIV, age, and sex were included as covariates for all correlation analyses. FDR‐corrected p values were provided. ACC, anterior cingulate cortex; EC, empathic concern; GMV, gray matter volume; ROIs, regions of interest. **p < .01.

Partial correlation analyses showed significant positive correlation between GMV in the left subgenual ACC and EC scores (*r* = .530, p = .006; Figure 2b). No significant correlations were observed between GMV in other empathy‐related ROIs and IRI subscales (all p > .05).

Furthermore, marginally significant positive correlation was observed between GMV in the left subgenual ACC and years with dance partners (*r* = .420, p = .087; Figure 2a). No significant correlations were observed between GMV in other empathy‐related ROIs and dance attributes (all p > .05).

### 3.3 Correlations between resting‐state FCs and behavioral variables

**FIGURE 3:**
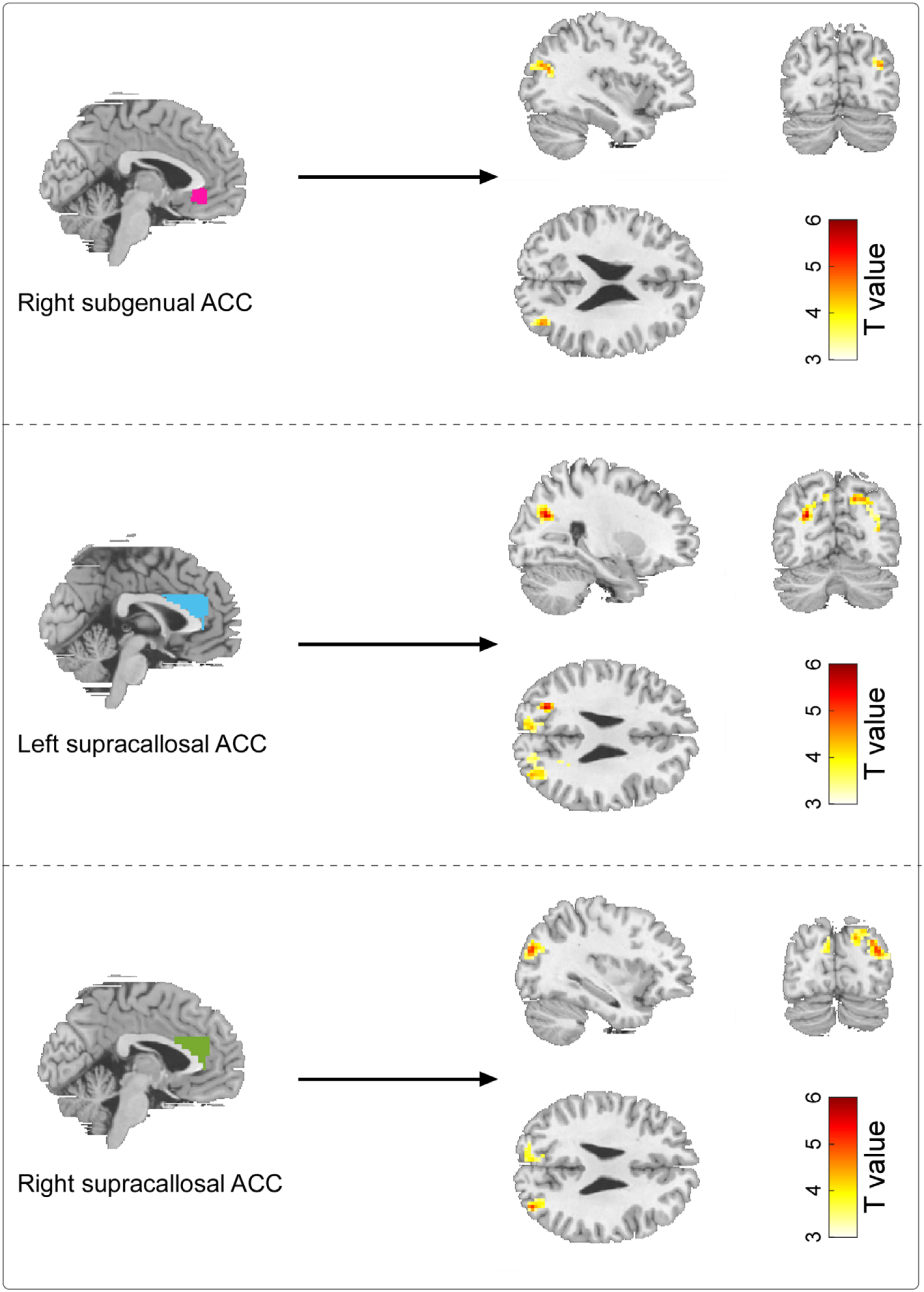
Correlations between resting‐state FCs of brain regions and EC scores for dancers. Resting‐state FCs between (1) the right subgenual ACC and right MOG (top), (2) the left supracallosal ACC and left and right SOG, MOG, and cuneus (middle), and (3) the right supracallosal ACC and left cuneus extending to SOG and right MOG extending to cuneus (bottom) showed significantly positive correlations with EC scores for dancers. Empathy‐related ROIs were displayed in the left column, and brain regions showed significant ROI‐based FCs with EC scores were showed in the right column. Mean FD, age, and sex were included as covariates for all correlation analyses. Clusters were considered as significant only if they reached a threshold of voxel‐level p < .001 and cluster‐level family‐wise error‐corrected p < .05.

For resting‐state fMRI data, FCs between the ACC and occipital gyrus showed positive correlations with EC scores for dancers (Figure 3). Specifically, strong evidence for significant correlations was observed for FCs between (1) the right subgenual ACC and right middle occipital gyrus (MOG), (2) the left supracallosal ACC and left MOG/left superior occipital gyrus (SOG), (3) the left supracallosal ACC and right SOG/right cuneus/right MOG, (4) the left supracallosal ACC and left SOG/left cuneus, (5) the right supracallosal ACC and right MOG/right cuneus, and (6) the right supracallosal ACC and left SOG/left cuneus (Table 2 and Figure 3).

**TABLE 2:** Resting‐state FCs of brain regions showed significant correlations with EC scores for dancers, and their correlations with years with dance partners

					Peak MNI coordinates			Correlations with years with partners	
Empathy‐related ROIs	Cluster size	Cluster p (FWE)	Brain regions	*t*	X	Y	Z	*r*	p_FDR_ value
Right subgenual ACC	45	.001	Right MOG (BA 19)	4.694	36	−75	24	.426	.023
			Right MOG (BA 19)	3.873	36	−84	21		
Left supracallosal ACC	59	<.001	Left MOG	5.607	−27	−69	27	.189	.255
			Left SOG	4.016	−21	−69	36		
			Left MOG	3.952	−24	−81	39		
	259	<.001	Right SOG (BA 19)	5.208	21	−87	33	.325	.069
			Right Cuneus	5.146	15	−81	36		
			Right MOG	5.008	30	−75	30		
	92	<.001	Left SOG (BA 31)	4.692	−15	−75	24	.273	.117
			Left Cuneus	4.513	−9	−81	24		
			Left SOG	4.503	−12	−87	30		
Right supracallosal ACC	158	<.001	Right MOG	5.064	33	−81	27	.426	.023
			Right Cuneus	4.589	15	−81	36		
			Right Cuneus	4.424	21	−87	33		
	56	.007	Left SOG	4.719	−12	−87	30	.370	.044
			Left Cuneus	4.116	−12	−75	24		

*Notes*: Clusters were considered significant only if they reached a threshold of voxel‐level p < .001 and cluster‐level FWE‐corrected p < .05. Mean FD, age, and sex were included as covariates for all correlation analyses. FDR‐corrected p values were provided.

Abbreviations: ACC, anterior cingulate cortex; BA, broadman area; EC, empathic concern; FC, functional connectivity; FWE, family‐wise error; MNI, Montreal Neurological Institute; MOG, middle occipital gyrus; ROIs, regions of interest; SOG, superior occipital gyrus.

**FIGURE 4:**
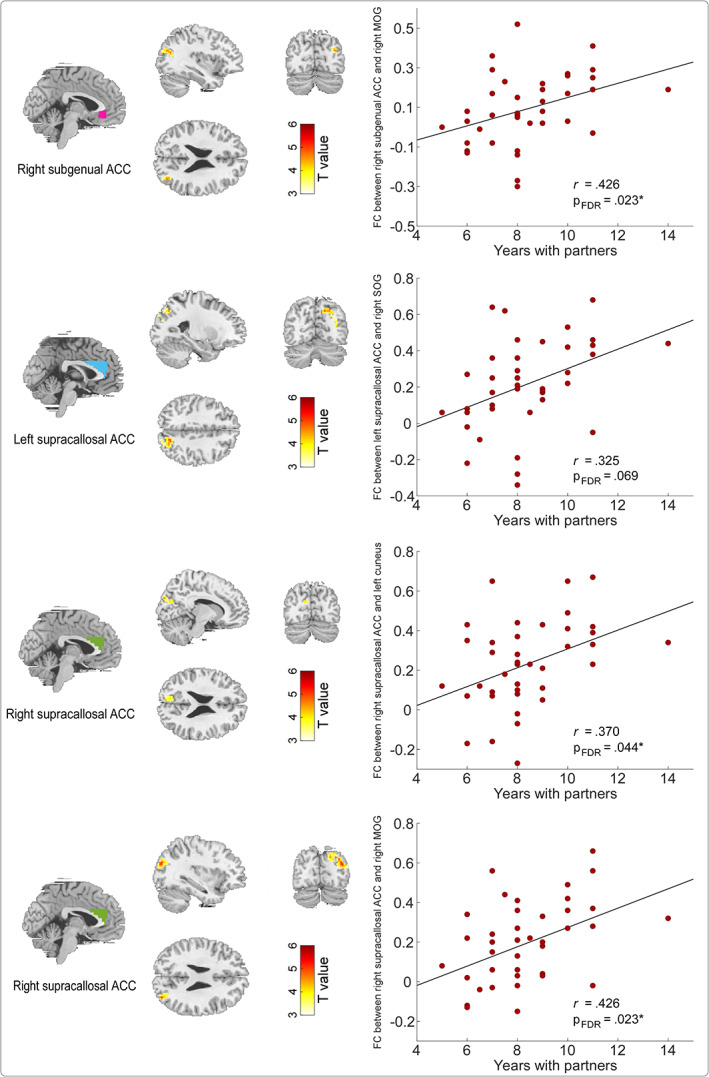
ROI‐based FCs showed significant correlations with EC scores also significantly correlated with years with dance partners for dancers. ROI‐based FCs showed significant correlations with EC scores were displayed in the left column, and correlations between these ROI‐based FCs and years with dance partners were shown in the right column. Resting‐state FCs between (1) the right subgenual ACC and right MOG, (2) the left supracallosal ACC and right SOG, (3) the right supracallosal ACC and left cuneus, and (4) the right supracallosal ACC and right MOG were displayed from top to bottom. Mean FD, age, and sex were included as covariates for all correlation analyses. FDR‐corrected p values were provided. ACC, anterior cingulate cortex; FC, functional connectivity; MOG, middle occipital gyrus; SOG, superior occipital gyrus. *p < .05.

Moreover, ROI‐based FCs showing significant correlations with EC scores were also positively correlated with years with dance partners (Figure 4). Specifically, clear evidence for significant correlations with years with dance partners was observed for (1) FCs between the right subgenual ACC and right MOG (*r* = .426, p = .023), (2) FCs between the right supracallosal ACC and right MOG extending to cuneus (*r* = .426, p = .023), and (3) FCs between the right supracallosal ACC and left cuneus extending to SOG (*r* = .370, p = .044; Figure 4). In addition, marginally significant correlation with years with dance partners was observed for FCs between the left supracallosal ACC and right SOG extending to cuneus and MOG (*r* = .325, p = .069).

To rule out possible confounding factors in the above correlation analyses, we conducted a series of partial correlation analyses (1) between EC scores and ACC‐related FC, and (2) between years with dance partners with ACC‐related FC for dancers. As summarized in Tables S4 and S5, ACC‐related FC (i.e., between the right subgenual ACC and right MOG; between the right supracallosal ACC and right MOG extending to cuneus; and between the right supracallosal ACC and left cuneus extending to SOG) was robustly and significantly correlated with EC scores and years with dance partners when the collected variables were included as covariates (i.e., sex, age, mean FD, years of the longest romantic relationship, the number of romantic relationships, quality of romantic relationships, Big 5 Inventory‐2 subscale scores, and Interpersonal Competence subscale scores). Moreover, resting‐state FCs of brain regions showed significant correlations with EC scores for controls were summarized in Table S6.

### 3.4 Resting‐state FCs mediate the relationship between years with dance partners and EC scores

**FIGURE 5:**
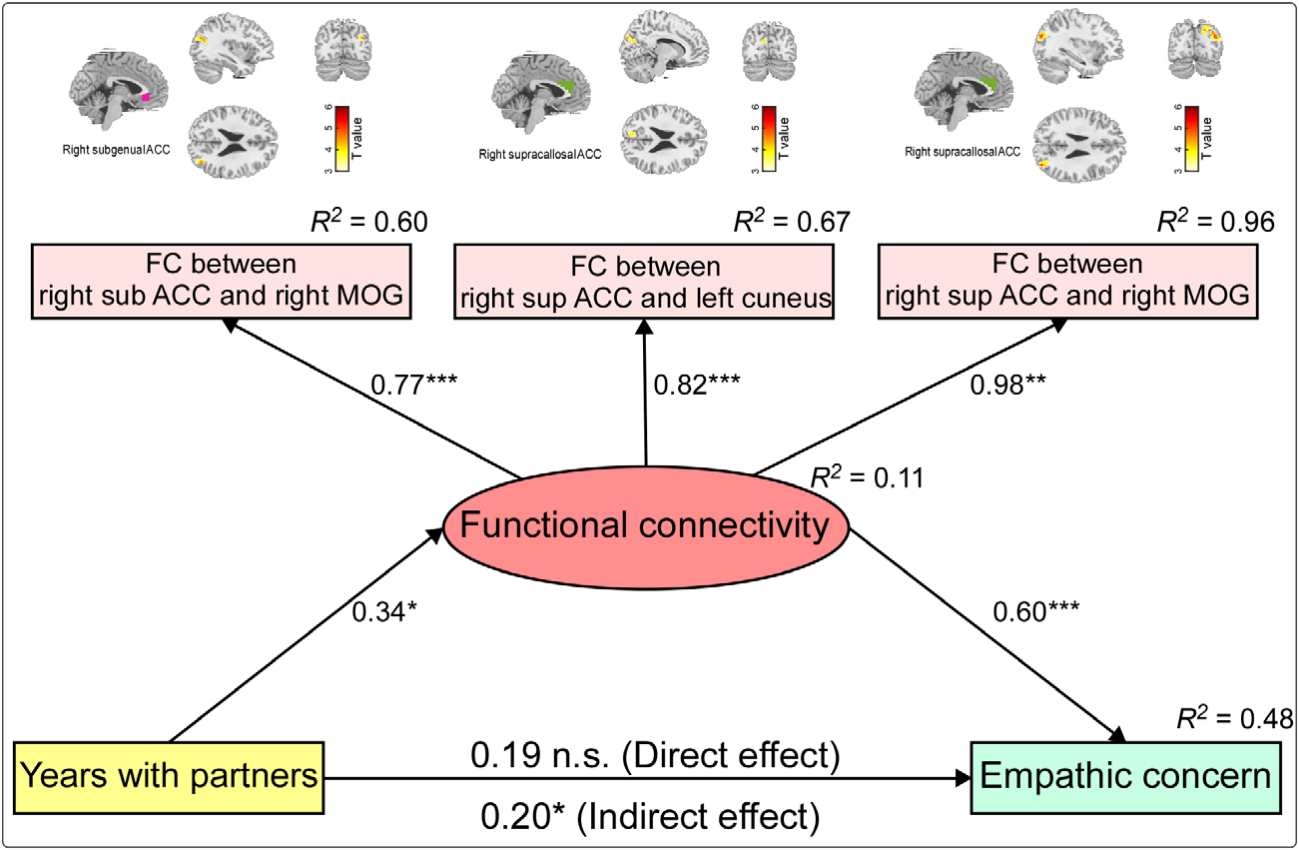
The mediation effect of resting‐state FCs on the relationship between years with dance partners and EC scores. The relationship between years with dance partners and EC scores was indirect‐only mediated by resting‐state FCs, which were loaded by FCs between the right subgenual ACC and right MOG, the right supracallosal ACC and left cuneus, as well as the right supracallosal ACC and right MOG. Standardized regression weights and squared multiple correlation coefficients were shown for the model. ACC, anterior cingulate cortex; FC, functional connectivity; MOG, middle occipital gyrus; n.s., not significant; *p < .05; **p < .01; ***p < .001.

ROI‐based resting‐state FCs could reliably mediate the relationship between years with dance partners and EC scores (Figure 5). Specifically, ROI‐based FCs were the mediators and loaded by FCs between the right subgenual ACC and right MOG, the right supracallosal ACC and left cuneus, and the right supracallosal ACC and right MOG. The final model fit the data well and exhibited adequate fit indices: χ^2^(4) = 3.732, p = .345, χ^2^/df = 0.933, RMSEA < 0.001, GFI = 0.967, AGFI = 0.877, CFI = 1.000, NFI = 0.966. Years with dance partners demonstrated an indirect effect (*b* = 0.202, SE = 0.093, CI = [0.044, 0.411], p = .011), but not a direct effect (*b* = 0.193, SE = 0.116, CI = [−0.074, 0.396], p = .131) on EC scores through the ROI‐based FCs. These results suggested that the ROI‐based FCs were the indirect‐only mediators on the relationship between years with dance partners and EC scores.

## 4 DISCUSSION

In this study, we aimed to investigate the link between long‐term training of ballroom dance and empathy and explore the neural mechanisms underlying this phenomenon. We obtained four main findings. First, ballroom dancers exhibited higher EC toward others than their age, sex, and education level matched controls. More importantly, the EC scores were positively correlated with years with dance partners but negatively correlated with the number of dance partners for ballroom dancers, suggesting a strong link between ballroom dance training and EC. Second, the EC scores were positively correlated with the GMV in the subgenual ACC for ballroom dancers, providing the structural basis for the promoted EC associated with dance training. Third, resting‐state FCs between the ACC (i.e., subgenual and supracallosal ACC) and occipital gyrus (i.e., MOG, SOG, and cuneus) showed positive correlations with both EC scores and years with dance partners for ballroom dancers. Fourth, the relationship between years with dance partners and EC scores was indirect‐only mediated by resting‐state FCs between the ACC and occipital gyrus. More importantly, the association between ballroom dance training and EC scores could be independent of one's romantic relationship status, personality, and sociability. These findings improved our understanding of the neural basis underlying the close link between ballroom dance training and EC, and provided evidence supporting that as a type of duo dances, ballroom dance could be used as a training strategy to improve empathic ability in practice.

In the past few decades, dance training has been considered as a way to improve muscular strength and endurance, balance, and other aspects of functional fitness (Hwang & Braun, 2015), as well as a training program to preserve cognitive, motor, and perceptual abilities from degradation for the elderly (Kattenstroth et al., 2010). The therapeutic benefits of dance were also demonstrated in clinical populations, such as patients with Parkinson disease (de Natale et al., 2017; McNeely et al., 2015; Pereira et al., 2019) and dementia (Karkou & Meekums, 2017). In contrast, this study explored the benefit of dance training from a very different but fresh perspective.

EC, the desire to promote others' well‐being or alleviate their suffering (Davis, 1980; Zhao et al., 2021), is widely regarded as the trait to motivate costly altruism and prosocial behavior (Batson et al., 1987; Eisenberg et al., 1989). It is primarily motivated by feelings of empathic other‐oriented concern rather than by the urgency to reduce our own discomfort (FeldmanHall et al., 2015). In this study, we observed that ballroom dancers showed higher EC scores than controls and that the EC scores were positively correlated with years with dance partners but negatively correlated with the number of dance partners for ballroom dancers (Figure 1). These observations suggested that ballroom dancers are more likely to experience feelings of concern for others due to long‐term training with fixed dance partners.

These behavioral findings could be explained in two different aspects. First, as a type of athletic training, dance training involves motor imitation, which could improve empathic ability according to the motor theory of empathy (Leslie et al., 2004). Note that several lines of evidence across species revealed a strong link between dance and motor imitation (Laland et al., 2016). Due to long‐term dance training, dancers show increased motor resonance as they observe and simulate the other's action in daily practice and performance. Since motor imitation plays a central role in empathy (de Waal & Preston, 2017), the promoted EC observed in ballroom dancers might be built upon their superior motor imitation. Second, distinct from other athletic training or activities, dance training also involves artistic training, which is important to improve the ability of the empathetic understanding of others (Freedberg & Gallese, 2007). Indeed, ballroom dance is unique in terms of the way in which the two dancers interact with each other during the duo‐dance training. Notably, other athletic and artistic activity forms, such as yoga, have been suggested to be associated with significant enhancements in both EC and other empathy components, for example, PD and PT (Acevedo et al., 2021). The diverse results imply that the benefits of activities on empathy might be varied based on activity types, as the engagement of different types of activities requires different skills. When compared with the performers of activities that can be accomplished alone, ballroom dancers need to pay close attention to their dance partners during the whole dance and keep the head clear that they are different since they need to perform different body movements. Moreover, to improve the artistry of the dance, ballroom dancers need to understand the emotions and feelings of their dance partners empathetically. Therefore, the promoted EC in ballroom dancers might also be associated with their improved ability to understand their dance partners during the long‐term artistic training.

Our behavioral results were further supported by the brain structural and functional imaging data. Specifically, we observed that for ballroom dancers, the GMV in the subgenual ACC was positively correlated with the EC scores (Figure 2) and resting‐state FCs between the ACC (i.e., subgenual and supracallosal ACC) and occipital gyrus (i.e., MOG, SOG, and cuneus) were positive correlations with both EC scores (Figure 3) and years with dance partners (Figure 4). The subgenual ACC is a brain area highly associated with regulating emotional responses, making it a prime region to directly mediate the helping behavior (Decety & Cacioppo, 2011). The activity in the subgenual ACC was also reported to support the relationship between EC and costly altruism (FeldmanHall et al., 2015). Similarly, the supracallosal ACC, or named dorsal ACC or anterior midcingulate cortex (Rolls, 2019), plays a crucial role in EC, and it is consistently activated in various tasks involving empathy (Fan et al., 2011). This claim was strongly supported by evidence from a lesion study, in which monkeys showed reduced prosocial preferences with ACC lesions, including the subgenual and supracallosal ACC (Basile et al., 2020). In contrast, the MOG and cuneus were often reported to be correlated with the processing of attentional visuospatial information (Baltaretu et al., 2021; Rotshtein et al., 2007), which would be more related to the dance training, instead of empathy improvement *per se* in this study. A previous study on early blind showed that the right MOG showed a preference for spatial over nonspatial processing stimuli, and the MOG activity was correlated with the accuracy of individual sound localization performance (Renier et al., 2010). The right cuneus was also activated by both visual search and memory search, which was responsible for both spatial and nonspatial shifts of attention, including attentional shifts in long‐term memory (Holzschneider et al., 2012; Makino et al., 2004; Weidner et al., 2002). Besides, cuneus was involved in the bottom‐up control of spatial attention (Hahn et al., 2006). Ballroom dance involves a lot of visuospatial information processing to perform enthusiastically body movements, such as head swing, body bent, and body rotation, and these movements and rotations can be seen in almost all types of ballroom dance type, such as foxtrot, waltz, swing, tango, rumba, samba, and cha‐cha. Therefore, it would be reasonable that the MOG and cuneus are functionally involved in the attentional visuospatial information processing improved during ballroom dance training. Since the ACC (i.e., subgenual and supracallosal ACC) and occipital gyrus (i.e., MOG, SOG, and cuneus) are involved in different functions, their association could be important to improve our understanding of the effect of long‐term ballroom dance training on empathy ability. Indeed, the mediation model showed that resting‐state FCs between the ACC and occipital gyrus (i.e., between the right subgenual ACC and right MOG, between the right supracallosal ACC and left cuneus extending to SOG, as well as between the right supracallosal ACC and right MOG extending to right cuneus) subserved an indirect‐only mediation effect on the relationship between years with dance partners and EC scores (Figure 5).

## 5 CONCLUSION

In summary, we provided solid behavioral and neural evidence showing that long‐term ballroom dance training with relatively fixed dance partners is associated with one's empathic concern. Theoretically, our study deepens our understanding of the neural mechanisms underlying the link between ballroom dance training and EC, and highlights the crucial role of resting‐states FCs between the ACC and occipital gyrus in mediating the relationship between dance training and EC. Practically, our study shed new insight into the development of duo dance‐based programs to improve empathic ability, thus helping people with impaired empathy, such as individuals with schizophrenia or autism spectrum disorder. Crucially, our results indicate that the enhanced EC in dancers could be due to the long‐term training with their dance partners, rather than other inherent differences, as no differences in personality or sociability were found between the two groups. It should be noted that our conclusions were drawn based on correlational results, and longitudinal studies with tasks measuring empathic behaviors are needed in further investigations to demonstrate a causal link between duo dance training and the development of the empathic brain.

## CONFLICT OF INTEREST

The authors declare that there is no conflict of interest that could be perceived as prejudicing the impartiality of the research reported.

## Supporting information


**TABLE S1** Descriptive statistics (*M* ± SD) of information about daily exercise and romantic relationships for dancers and controls.
**TABLE S2** Descriptive statistics (*M* ± SD) of subscale scores in the Big 5 Inventory‐2 and the Questionnaire of Interpersonal Competence for dancers and controls.
**TABLE S3** Correlation results between EC scores and subscale scores in the Big 5 Inventory‐2 and the Questionnaire of Interpersonal Competence using Pearson Correlation analyses and partial correlation analyses with age and sex as covariates.
**TABLE S4** Partial correlation analyses between EC scores and ACC‐related functional connectivity.
**TABLE S5** Partial correlation analyses between years with dance partners and ACC‐related functional connectivity.
**TABLE S6** Resting‐state FCs of brain regions showed significant correlations with EC scores for controls.

## Data Availability

The data in the present study are available upon reasonable request to the corresponding author.
