# Supplementary material for: The association between ballroom dance training and empathic concern: Behavioral and brain evidence
Source: Hum Brain Mapp. 2022 Aug 16;44(2):315–26. doi: 10.1002/hbm.26042 (PMC9842917; doi:10.1002/hbm.26042)
Supplement: Supplementary file 1 — TABLE S1 Descriptive statistics (M ± SD) of information about daily exercise and romantic relationships for dancers and controls. TABLE S2 Descriptive statistics (M ± SD) of subscale scores in the Big 5 Inventory‐2 and the Questionnaire of Interpersonal Competence for dancers and controls. TABLE S3 Correlation results between EC scores and subscale scores in the Big 5 Inventory‐2 and the Questionnaire of Interpersonal Competence using Pearson Correlation analyses and partial correlation analyses with age and sex as covariates. TABLE S4 Partial correlation analyses between EC scores and ACC‐related functional connectivity. TABLE S5 Partial correlation analyses between years with dance partners and ACC‐related functional connectivity. TABLE S6 Resting‐state FCs of brain regions showed significant correlations with EC scores for controls. [file HBM-44-315-s001.docx]

Table S1. Descriptive statistics (M ± SD) of information about daily exercise and romantic relationships for dancers and controls.

| Variables | Dancer group | Control group | χ^2^/U | *p* |
| --- | --- | --- | --- | --- |
|  | (n=41) | (n=40) |  |  |
| Like sedentary / engage in activities | 23/18 | 26/14 | 0.67 | 0.413 |
| Romantic Status (In a relationship/Not in a relationship) | 26/15 | 13/27 | 7.75 | 0.005 |
| Number of romantic relationships ever had | 2.22 ± 1.25 (n = 37) | 1.68 ± 1.58 (n = 37) | 867.50 | 0.042 |
| Years of the longest romantic relationship | 3.61 ± 2.13 (n = 36) | 1.80 ± 1.66 (n = 28) | 795.00 | < 0.001 |
| Quality of romantic relationships | 8.90 ± 1.60 (n = 39) | 7.32 ± 2.09 n = 31) | 867.00 | 0.002 |

Note #1: One participant in the dancer group had eight years of regular swimming activity. One participant in the control group had two years of regular running activity, one in the control group had two years of regular basketball playing activity, and one in the control group had three years of regular self-weighing training. Others had no regular exercise habit of training for more than a year.

Note #2: Seven participants (four in the dancer group and three in the control group) were unwilling to report the number of romantic relationships they ever had. Six participants (three in the dancer group and three in the control group) were unwilling to report years of the longest romantic relationships, and 11 participants (two in the dancer group and nine in the control group) reported that they had never had a romantic relationship before. These 11 participants did not report years of the longest romantic relationship and quality of romantic relationships.

Table S2. Descriptive statistics (M ± SD) of subscale scores in the Big 5 Inventory-2 and the Questionnaire of Interpersonal Competence for dancers and controls.

| Variables | Dancer group | Control group | *t* | *p* |
| --- | --- | --- | --- | --- |
|  | (n=41) | (n=40) |  |  |
| **Big 5 Inventory -2** |  |  |  |  |
| Extraversion | 39.17 ± 7.56 | 35.78 ± 6.96 | 2.10 | 0.128 |
| *Sociability* | 11.93 ± 3.23 | 10.58 ± 3.25 | 1.88 | 0.128 |
| Agreeableness | 46.80 ± 5.61 | 45.43 ± 6.21 | 1.05 | 0.398 |
| Conscientiousness | 44.66 ± 8.17 | 41.40 ± 6.50 | 1.98 | 0.128 |
| Negative emotionality | 35.51 ± 8.40 | 33.68 ± 9.13 | 0.94 | 0.398 |
| Open-mindedness | 43.78 ± 7.78 | 42.25 ± 8.41 | 0.85 | 0.398 |
| **Interpersonal competence** |  |  |  |  |
| Initiating relationships | 26.59 ± 5.00 | 25.55 ± 5.46 | 0.89 | 0.470 |
| Asserting displeasure with others' actions | 27.83 ± 4.14 | 25.78 ± 4.95 | 2.02 | 0.235 |
| Self-disclosure | 23.63 ± 4.19 | 24.85 ± 4.88 | -1.20 | 0.388 |
| Managing interpersonal conflicts | 26.88 ± 4.98 | 28.28 ± 4.52 | -1.32 | 0.388 |
| Providing emotional support | 31.73 ± 4.34 | 31.85 ± 3.98 | -0.13 | 0.899 |

*Note: all p values were corrected using an FDR procedure.*

Table S3. Correlation results between EC scores and subscale scores in the Big 5 Inventory-2 and the Questionnaire of Interpersonal Competence using Pearson Correlation analyses and partial correlation analyses with age and sex as covariates.

| Variables | Pearson correlation | | Partial correlation | |
| --- | --- | --- | --- | --- |
|  | *r* | *p* | *r* | *p* |
| **Big 5 Inventory -2** |  |  |  |  |
| Extraversion | 0.099 | 0.537 | 0.186 | 0.444 |
| *Sociability* | -0.016 | 0.921 | -0.003 | 0.985 |
| Agreeableness | 0.259 | 0.101 | 0.301 | 0.189 |
| Conscientiousness | 0.167 | 0.298 | 0.172 | 0.444 |
| Negative emotionality | 0.017 | 0.918 | -0.112 | 0.598 |
| Open-mindedness | 0.262 | 0.097 | 0.326 | 0.189 |
| **Interpersonal competence** |  |  |  |  |
| Initiating relationships | 0.152 | 0.341 | 0.290 | 0.370 |
| Asserting displeasure with others' actions | 0.176 | 0.270 | 0.208 | 0.510 |
| Self-disclosure | -0.102 | 0.527 | -0.016 | 0.922 |
| Managing interpersonal conflicts | -0.165 | 0.302 | -0.061 | 0.922 |
| Providing emotional support | 0.043 | 0.790 | 0.038 | 0.922 |

*Note: all p values were corrected using an FDR procedure.*

Table S4. Partial correlation analyses between EC scores and ACC-related functional connectivity.

| Covariates | Functional connectivity | *r* | *p* | *df* |
| --- | --- | --- | --- | --- |
| sex & age & mean FD | subgeneual ACC and right MOG | 0.649 | < 0.001 | 36 |
|  | supracallosal ACC and left cuneus | 0.600 | < 0.001 | 36 |
|  | supracallosal ACC and right MOG | 0.683 | < 0.001 | 36 |
| sex & age & mean FD & years of the longest romantic relationship | subgeneual ACC and right MOG | 0.630 | < 0.001 | 30 |
|  | supracallosal ACC and left cuneus | 0.628 | < 0.001 | 30 |
|  | supracallosal ACC and right MOG | 0.646 | < 0.001 | 30 |
| sex & age & mean FD & the number of romantic relationships | subgeneual ACC and right MOG | 0.630 | < 0.001 | 31 |
|  | supracallosal ACC and left cuneus | 0.619 | < 0.001 | 31 |
|  | supracallosal ACC and right MOG | 0.672 | < 0.001 | 31 |
| sex & age & mean FD & quality of romantic relationships | subgeneual ACC and right MOG | 0.615 | < 0.001 | 33 |
|  | supracallosal ACC and left cuneus | 0.574 | < 0.001 | 33 |
|  | supracallosal ACC and right MOG | 0.627 | < 0.001 | 33 |
| sex & age & mean FD & Big 5 Inventory-2 subscale scores | subgeneual ACC and right MOG | 0.661 | < 0.001 | 31 |
|  | supracallosal ACC and left cuneus | 0.542 | 0.002 | 31 |
|  | supracallosal ACC and right MOG | 0.624 | < 0.001 | 31 |
| sex & age & mean FD & Interpersonal Competence subscale scores | subgeneual ACC and right MOG | 0.687 | < 0.001 | 31 |
|  | supracallosal ACC and left cuneus | 0.583 | < 0.001 | 31 |
|  | supracallosal ACC and right MOG | 0.677 | < 0.001 | 31 |

*Note: all p values were corrected using an FDR procedure. Since some participants were unwilling to report some information, different variables were separately included as the covariates in the partial correlation analyses, and the degree of freedom was not the same in different analyses.*

Table S5. Partial correlation analyses between years with dance partners and ACC-related functional connectivity.

| Covariates | Functional connectivity | *r* | *p* | *df* |
| --- | --- | --- | --- | --- |
| sex & age & mean FD | subgeneual ACC and right MOG | 0.426 | 0.009 | 36 |
|  | supracallosal ACC and left cuneus | 0.370 | 0.022 | 36 |
|  | supracallosal ACC and right MOG | 0.426 | 0.009 | 36 |
| sex & age & mean FD & years of the longest romantic relationship | subgeneual ACC and right MOG | 0.500 | 0.004 | 30 |
|  | supracallosal ACC and left cuneus | 0.421 | 0.016 | 30 |
|  | supracallosal ACC and right MOG | 0.547 | 0.002 | 30 |
| sex & age & mean FD & the number of romantic relationships | subgeneual ACC and right MOG | 0.459 | 0.011 | 31 |
|  | supracallosal ACC and left cuneus | 0.374 | 0.032 | 31 |
|  | supracallosal ACC and right MOG | 0.449 | 0.011 | 31 |
| sex & age & mean FD & quality of romantic relationships | subgeneual ACC and right MOG | 0.477 | 0.005 | 33 |
|  | supracallosal ACC and left cuneus | 0.380 | 0.025 | 33 |
|  | supracallosal ACC and right MOG | 0.500 | 0.003 | 33 |
| sex & age & mean FD & Big 5 Inventory-2 subscale scores | subgeneual ACC and right MOG | 0.438 | 0.016 | 31 |
|  | supracallosal ACC and left cuneus | 0.384 | 0.027 | 31 |
|  | supracallosal ACC and right MOG | 0.416 | 0.019 | 31 |
| sex & age & mean FD & Interpersonal Competence subscale scores | subgeneual ACC and right MOG | 0.473 | 0.008 | 31 |
|  | supracallosal ACC and left cuneus | 0.386 | 0.027 | 31 |
|  | supracallosal ACC and right MOG | 0.424 | 0.017 | 31 |

*Note: all p values were corrected using an FDR procedure. Since some participants were unwilling to report some information, different variables were separately included as the covariates in the partial correlation analyses, and the degree of freedom was not the same in different analyses.*

Table S6. Resting-state FCs of brain regions showed significant correlations with EC scores for controls.

|  |  |  |  |  | Peak MNI coordinates | | |
| --- | --- | --- | --- | --- | --- | --- | --- |
| Empathy-related ROIs | cluster size | Cluster *p* (FWE) | Brain regions | *t* | X | Y | Z |
| Left insula | 38 | 0.002 | Right insula | 5.050 | 42 | 3 | -9 |
| Right supracallosal ACC | 76 | < 0.001 | Right ITG | 4.724 | 57 | -48 | -27 |
|  |  |  | Right MTG | 4.259 | 51 | -36 | -12 |
|  |  |  | Right MTG | 4.189 | 66 | -27 | -18 |

*Note: clusters were considered significant only if they reached a threshold of voxel-level p < 0.001 and cluster-level family-wise error (FWE) corrected p < 0.05. Mean FD, age, and sex were included as covariates for all correlation analyses. FC, functional connectivity. ROIs, Regions of Interest; MNI, Montreal Neurological Institute; EC, Empathic Concern; ACC, Anterior Cingulate Cortex; ITG, Inferior Temporal Gyrus; MTG, Middle Temporal Gyrus.*
